# Occurrence and Succession of Bacterial Community in O_3_/BAC Process of Drinking Water Treatment

**DOI:** 10.3390/ijerph16173112

**Published:** 2019-08-27

**Authors:** Sheng Dong, Lijun Liu, Yuxiu Zhang, Fajun Jiang

**Affiliations:** 1 School of Chemical and Environmental Engineering, China University of Mining and Technology (Beijing), Beijing 100083, China; 2 Shenzhen Water Group, Shenzhen 518060, China; 3 Guangxi Key Laboratory of Marine Environmental Science, Beibu Gulf Marine Research Center, Guangxi Academy of Sciences, Nanning 530007, China

**Keywords:** O_3_/BAC, bacterial community, diversities and dynamics, succession procedure

## Abstract

In the drinking water industry, a common advanced treatment process is comprised of treatment with ozone, followed by biological-activated carbon (O_3_/BAC). However, the bacterial community formation and succession procedures associated with activated carbon have rarely been reported. In this study, the dynamics of bacterial communities at three different depths were investigated using a pilot-scale O_3_/BAC filter. The average chemical oxygen demand (CODMn), turbidity removal and dissolved oxygen (DO) consumption rate of the filter were 26.43%, 16.57% and 16.4% during the operation period, respectively. Bacterial communities dominated by proteobacteria and Bacteroidetes attached on activated carbon were determined by polymerase chain reaction-density gradient gel electrophoresis (PCR-DGGE). Principal component analysis (PCA) revealed that the compositions and structures of bacterial communities in different layers clustered after fluctuation. A redundancy analysis (RDA) indicated that *Ramlibacter henchirensis* was positively correlated to chemical oxygen demand (COD_Mn_) removal and nitrate-N removal, and *Georgfuchsia toluolica* also showed a positive correlation with COD_Mn_ removal. *Aquabacterium parvum* and *Phaeobacterium nitratireducens* were positively-correlated with turbidity removal. *Pedobacter glucosidilyticus* and *Pseudomonas sp.* were associated with high dissolved oxygen (DO) consumption. These results provide insight into the succession characteristics of the bacterial community of O_3_/BAC treatment and the interactions of the bacterial community with filter operation performance.

## 1. Introduction

Installation of a traditional biological activated carbon (BAC) filter is a common practice that is widely used in drinking water treatment. It is an effective method for removing dissolved organic carbon (DOC) and controlling disinfection by-products (DBPs) [1,2,3]. The bacterial community associated with activated carbon plays a key role in biodegradation, and organic compounds are utilized to maintain the growth and reproduction of microorganisms [4,5]. To strengthen the biodegradability and improve the removal effects of ever-changing organic pollution, ozone is introduced and combined with the BAC process. Ozone is a reliable option for removing trace organic contaminants (TOrCs). TOrCs can be detected in ground water and surface water, and have facilitated increased public awareness in recent decades [6,7,8]. With strong oxidation, ozone can increase the level of biodegradable dissolved organic carbon (BDOC) with low molecular weight <1 kDa, which can then be effectively removed by the BAC filter [9,10,11].

Research has been conducted on the community structure of the BAC process, to investigate its functional characteristics and biological safety. Proteobacteria, Acidobacteria, Bacteroidetes, Cyanobacteria, and Planctomycetes were common bacterial phyla in the BAC filter. The phylum of Proteobacteria had the highest relative abundance, especially in filter effluent [12,13,14,15,16]. However, potential pathogens of genera *Chromobacterium* and *Sphingomonas* were detected in the drinking water biofilters [12]. Moreover, the leakage of opportunistic pathogens (OPs) and their proliferation in drinking water distribution systems (DWDSs) has become an emerging area of concern [17,18,19]. 

The O_3_/BAC process has been used in late stages of drinking water treatment in urban regions of southern China for years, and the expected effects have been achieved [10]. However, the knowledge regarding the occurrence, succession, and maturation procedures of the bacterial community is still very limited. It is necessary to investigate the formation of the associated biomass and its correlation with hydrothermal local environmental factors to improve the fundamental understanding of the O_3_/BAC process and guide parameter settings, as well as risk assessment. In this study, a pilot-scale filter for an O_3_/BAC process was set up to investigate the formation of the bacterial community attached to activated carbon in the long term, using a 16S rRNA-based polymerase chain reaction-density gradient gel electrophoresis (PCR-DGGE) method. Moreover, this study investigated the performance of the filter under dynamic changes of the classified bacteria.

## 2. Materials and Methods

### 2.1. Experimental Setup

**Figure 1:**
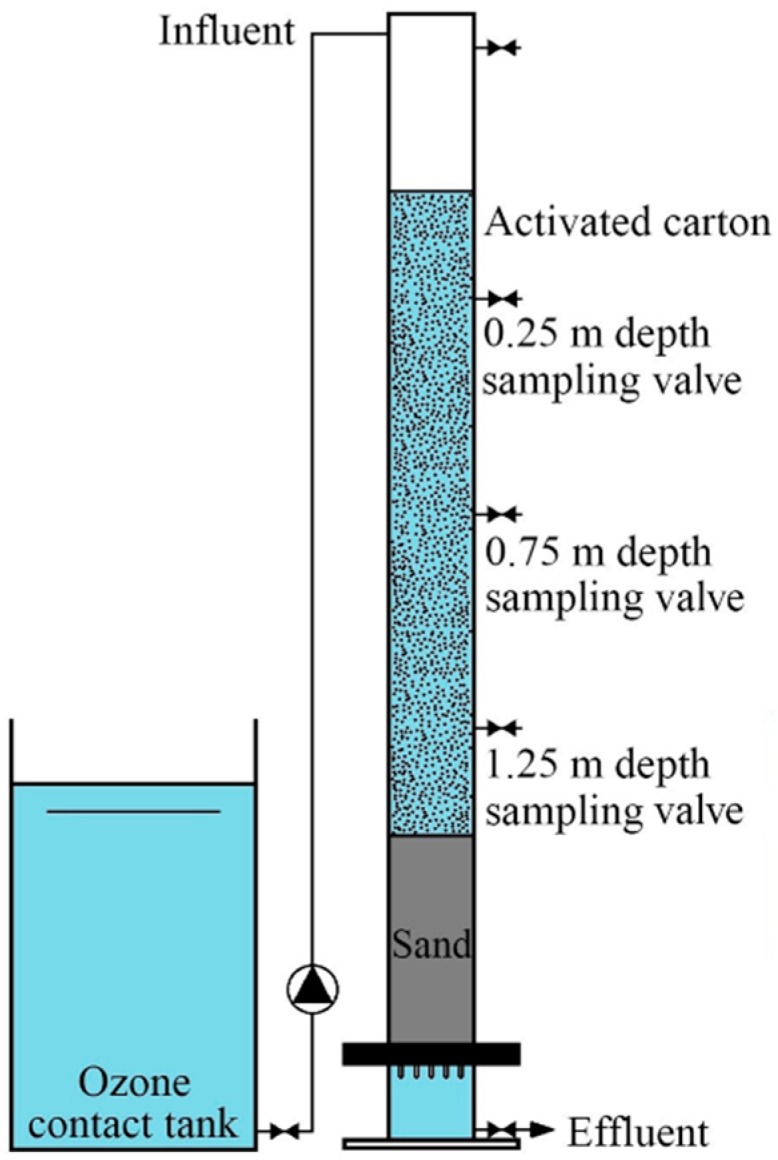
Experimental setup of the pilot-scale O_3_/biological activated carbon (BAC) filter.

A Plexiglass cylinder (200 mm diameter and 2.6 m length) filtration column was constructed and filled with a 200 mm supporting layer, a 500 mm sand layer (0.4–0.8 mm particle size), and a 1500 mm cracked activated carbon layer (0.9–1.1 mm particle size) from bottom to top, with reference to a typical water plant in Shenzhen (shown in Figure 1). Sampling ports for activated carbon were equipped at the 0.25 m, 0.75 m, and 1.25 m depths of the cracked activated carbon layer. The specification of the cracked activated carbon is listed in Table 1. The intake was at the top and the outlet was at the bottom. The pilot-scale filter was fed with the effluent of an ozonation contact reactor from the same water plant, with an ozone concentration of 5.0–10.0 mg/L and a hydraulic loading of 8 m/h. The filter operated for 360 days and no backwash was conducted for the maturation of the biomass.

### 2.2. Water Quality Analysis

Influent and effluent water samples of the pilot-scale filter were collected to analyze water quality parameters, including chemical oxygen demand (COD_Mn_), dissolved oxygen (DO), nitrate nitrogen, turbidity, temperature, and pH. The COD_Mn_ was determined using an acidic potassium permanganate method, and the nitrate nitrogen was determined using the phenol disulfonic acid spectrophotometry method described in "Water and Wastewater Quality Monitoring and Analysis Methods" [20]. The DO, pH, and temperature were measured using a hand-held dissolved oxygen meter 550A (YSI, Yellow Springs, Ohio, USA).

### 2.3. Bacterial Community Analysis

Activated carbon samples were collected from the 0.25 m, 0.75 m, and 1.25 m depths of the activated carbon layer at 30, 60, 120, 180, 240, and 360 days. Bacteria associated on the activated carbon was separated using an ultrasonic elution method [21,22]. In addition, 100 mL of sterile water was added to the 10 g activated carbon sample, which was then placed in a 250 mL conical flask and sonicated at 180 W and 55 KHz for 6 minutes. A solution of ultrasonic elution was collected. This elution procedure was repeated three times. Then, the 300 mL elution solution was filtrated using a 0.22 μm nitrocellulose filter, and the total DNA of the bacterial community was extracted using a “Water DNA kit” (Sigma). The extracted DNA was stored at −20 °C until further processing.

PCR amplifications of the bacterial 16S rRNA V3 region for DGGE were performed using a forward primer with a guanine–cytosine (GC) clamp at its 5′ end (5-CGCCCGCCGCGCGCGGCGGGCGGGGCGGGGGCACGGGGGCCTACGGGAGGCAGCAG-3′) and a reverse primer (5′-ATTACCGCGGCTGCTGG-3′) in a temperature gradient thermocycler. Each 40 μL reaction contained the following components: 20 μL of 2 × ExTaq DNA polymerase mixture (Takara, Japan), 0.25 μM of each primer, and 1 μL of the total DNA template. The PCR program was carried out with an initial denaturation step of 94 °C for 10 min, followed by 21 cycles of denaturation at 94 °C for 30 s, temperature gradient annealing from 65 to 55 °C for 30 s, and elongation at 72 °C for 30 s. This was followed by another 10 cycles of denaturation at 94°C for 30 s, annealing at 55°C for 30 s, and elongation at 72 °C for 30 s. The cycling was completed with a final elongation stage at 72 °C for 10 min. The lengths of the PCR products were verified by agarose gel (1.5%) electrophoresis.

The DGGE of the PCR-amplified 16S rRNA V3 region was performed using the “Dcode Universal Mutation Detection System” (Bio-Rad). The denaturing gradient gel was prepared with 10% (w/v) polyacrylamide and a 35–70% denaturing gradient, and was run for 12 h in a 1 × TAE buffer at a constant voltage of 75 V. After electrophoresis, the gel was stained for 20 min by GelRed (Biotium, Fremont, California, USA), and was photographed by the Gel DOC^TM^ XR + system (Bio-Rad, Hercules, California, USA). The prevalent DGGE bands were carefully excised and placed in 1.5 mL centrifuge tubes with a numbered order. In addition, 100 μL of sterile water was added to each tube, and DNA fragments were leached out overnight at 4 °C. Prevalent bands were verified by PCR-amplification and DGGE as previously described, and were excised for the second time. After confirmation, the leached DNA fragments of the second excised band were amplified and sequenced by BGI Shenzhen (China).

The sequenced results of this study were submitted to GeneBank under the accession numbers (MH973208) to (MH973221). The obtained sequences were blasted in the National Center for Biotechnology Information (NCBI) 16S ribosomal RNA sequence database in order to determine the species’ identities or closest relatives.

The electrophoregram of the DGGE was processed by “Quantity One” software (Bio-Rad, Hercules, California, USA). Each band in the same horizontal position presented an operational taxonomic unit (OTU). The diversity of bacterial community was assessed by the Shannon index (H′) [23,24], calculated for each sample using the following formula:

(1) $$H^{\prime }=-\sum_{i=1}^{R}p_{i}\ln p_{i}$$

where *p_i_* is often the proportion of individuals belonging to the species *i*, and *R* is the number of species found in the community.

## 3. Results

### 3.1. Biological Activated Carbon (BAC) Filter Performance

**Figure 2:**
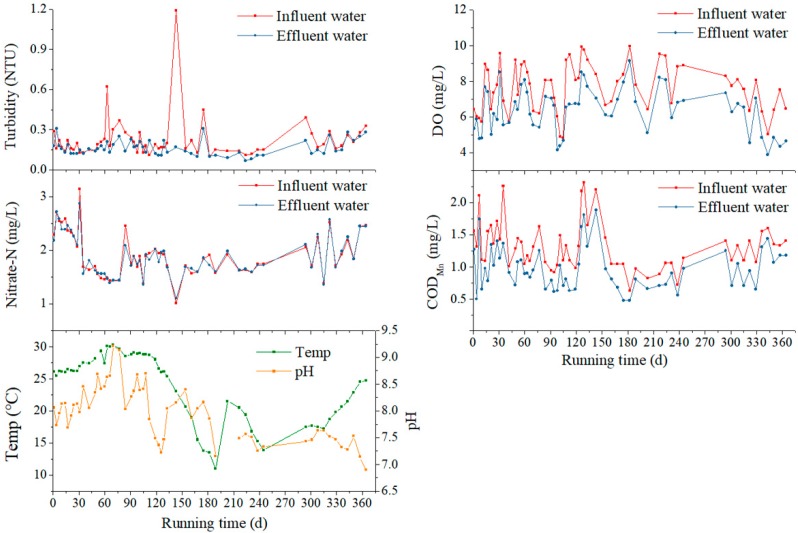
Influent and effluent water parameters of the BAC filter in 360 days.

The pilot-scale BAC filter in this study operated over 360 days. Parameters including COD_Mn_, DO, turbidity, nitrate nitrogen concentrations of influent and effluent water, as well as the pH and temperature of effluent water, were continuously determined. As revealed in Figure 2, the COD_Mn_ of effluent water was between 0.48 to 1.88 mg/L (mean value 0.97 mg/L), and that of influent water was between 0.63 to 2.31 mg/L (mean value 1.31 mg/L). The COD_Mn_ was effectively reduced by the pilot-scale filter, with an average removal rate of 26.43%. The turbidity of influent water fluctuated in a range from 0.10 to 1.19 nephelometric turbidity units (NTU) (mean value 0.22 NTU), whereas the range of the effluent water turbidity was evidently narrowed, as the concentration varied from 0.07 to 0.31 NTU (mean value 0.16 NTU). The average removal rate of turbidity was 16.57%. However, an increase of turbidity was observed occasionally, and might have been caused by a leakage of activated carbon powder or bio flocs. 

The DO of the influent water was between 4.84 to 9.96 mg/L (mean value 7.64 mg/L), and that of the effluent water samples remained in a concentration range between 3.88 to 9.14 mg/L (mean value 6.38 mg/L). This was adequate for microbial growth in the different activated carbon layers, and the microorganisms consumed an average of 16.4% of the DO. The relatively high concentration of DO caused a series of differences from a traditional BAC process. The levels of the nitrate nitrogen of influent and effluent water samples were almost identical during the whole procedure, indicating that there was no significant denitrification under the high-DO condition.

Owing to the adsorption of activated carbon and microbial metabolism, the BAC filter achieved an effective performance from the set-up period, as compared with reported data [25]. Referring to the GB5749-2006 standards for drinking water quality listed in Table 2, the main parameters of effluent water, including COD_Mn_, nitrate nitrogen, and turbidity, met the requirements. Moreover, after the fluctuation stage, the pH value gradually decreased into the required range.

### 3.2. Bacterial Community Analysis

#### 3.2.1. Polymerase Chain Reaction-Density Gradient Gel Electrophoresis (PCR-DGGE) and Bacteria Identification

**Figure 3:**
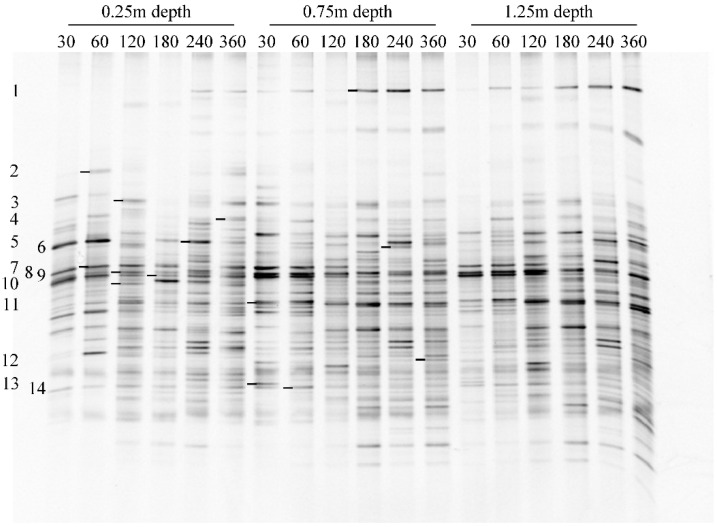
Density gradient gel electrophoresis (DGGE) fingerprints of bacterial communities attached to different depths of activated carbon layers in 360 days.

The bacterial communities associated with different samples from the three activated carbon layers in 360 days were analyzed by PCR-DGGE, and the electrophoregram is shown in Figure 3. Each band in the same horizontal position presented an OTU, and 50 types of OTUs were accordingly detected in total. Additionally, 14 dominant OTUs were successfully sequenced. Then, the sequences were blasted to identify the species or closest relatives in GeneBank database. Based on the blast results (listed in Table 3), proteobacteria was determined to be the predominant phylum of the bacterial community in the BAC filter, including 9 betaproteobacteria and 3 gammaproteobacteria. A total of 2 species Bacteroidetes were detected.

#### 3.2.2. Bacterial Community Diversities and Dynamics

The changes in OTU quantity and Shannon index, representing the diversity of the bacterial communities, are summarized in Table 4. Remarkably, an intense rise was observed in the OTUs and Shannon index of all the activated carbon layers. The layer at 1.25 m increased first, followed by the layers at 0.75 and 0.25 m depths in order. In addition, the OTU quantities and Shannon index values of the 0.75 m and 1.25 m depth layers were generally higher than the OTU quantity of the 0.25 m depth layer (except for the sample of day 360). These results indicated that the lower activated carbon layer was more conducive to the maturation of the bacterial community and a higher diversity under sufficient DO.

**Figure 4:**
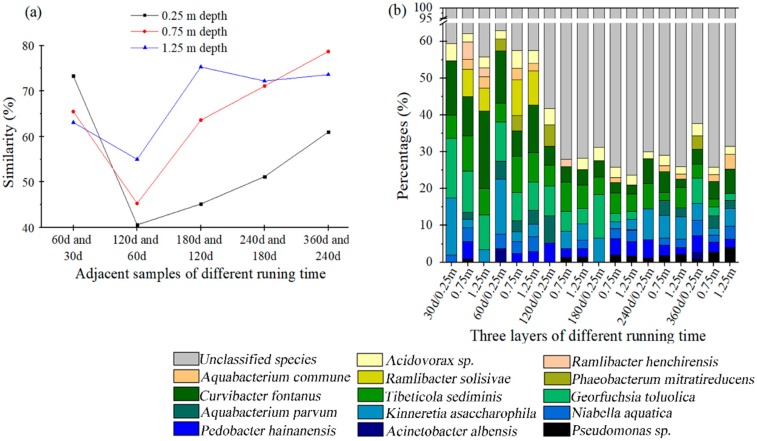
Similarity changes between time-adjacent samples from different carbon layers (**a**), relative abundances of bacterial species attached on different activated carbon samples (**b**).

The similarity in adjacent samples was introduced to describe the dynamics of the bacterial communities between time-adjacent samples in the same carbon layer. The similarity was analyzed by the Quantity One software based on a contrast of OTU types and the relative abundance in both adjacent samples. As revealed in Figure 4a, a significant decrease was noticed in the similarity between the samples of day 60 to day 120, indicating a serious shift of bacterial communities in all of the carbon layers during this period. The similarity of the 0.25 m depth layer fluctuated the most among the three active carbon layers, suggesting that the lower layer was advantageous for the stability of the bacterial community.

To get an insight into the structural dynamics of the bacterial communities attached to different activated carbon layers during the study, Figure 4b summarizes the percentages of the 14 sequenced dominant species and unclassified species during the 360 days. The predominant species at day 30 were *Curvibacter fontanus*, *Kinneretia asaccharophila*, *Tibeticola sediminis,* and *Georgfuchsia toluolica* from orders burkholderiales and nitrosomonadales, and these accounted for 52.8%, 33.8%, and 41.1% of the communities associated on the 0.25 m, 0.75 m, and 1.25 m depth carbon layers, respectively. As the study progressed and the diversity of the bacterial community increased, the total abundances of these species were constantly diluted, and decreased to 19.3% (0.25 m), 11.3% (0.75 m), and 13.4% (1.25 m) in the samples at day 360. The percentages of *Ramlibacter solisilvae* and *Ramlibacter henchirensis* were also decreased, and finally vanished after day 120. As oxyanion-reducing, non-flagellated, and slow-growing betaproteobacteriuia of genus *Ramlibacter* [26,27], both species presented at lower layers (0.75 and 1.25 m depth) with less fluid impact, and eventually disappeared because of competition. Conversely, *Pseudomonas sp.* and *Pedobacter glucosidilyticus* were enriched during the study. They appeared first in the layer at 0.75 m at day 30, and were further colonized to other layers. As a phototrophic bacterium [28], *Phaeobacterium nitratireducens* mainly presented at the surface layer. In contrast, *Aquabacterium commune* presented only at lower layers (0.75 and 1.25 m depths), rather than the species of *Ramlibacter*.

#### 3.2.3. Principal Component Analysis (PCA) and Redundancy Analysis (RDA)

**Figure 5:**
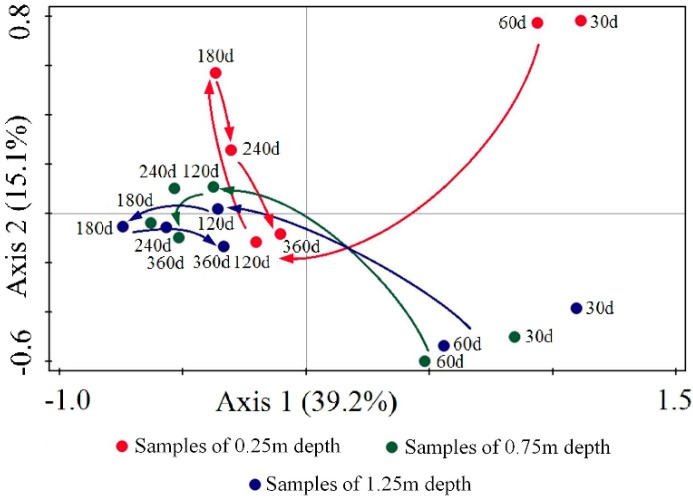
PCA scatter plot of the different samples in 360 days.

Principal component analysis (PCA) was performed based on the OTU type and relative abundance of different samples, as shown in Figure 5. The first principal component (PC1) and the second principal component (PC2) respectively explained 39.2% and 15.1% of the total variance.

The ordination plot of the PCA revealed the routes for different depths. The 0.25 m depth started at the first quadrant and moved down-leftward at day 120, then straightened and moved upward in the PC2 at day 180, and finally went down to the negative axis of PC1 at day 360. The 0.75 and 1.25 m depths both started at the fourth quadrant and moved up-leftward at day 120. Then, the 1.25 m depth continued moving leftward in the PC1 at day 180, and went rightward at days 240 and 360. However, the 0.75 m depth moved slightly and irregularly.

In investigating the mechanisms behind the PCA, the main reasons were determined to be the accumulation of competitive and dominant bacteria, and the different reactions of the three activated carbon layers after environmental influences. Although separated at the beginning, all three depths clustered with a similar bacterial community structure as early as day 120 (after a series of shifts). Based on the subsequent environmental disturbance, the bacterial consortium of each depth responded in different ways, and recovered at day 360.

**Figure 6:**
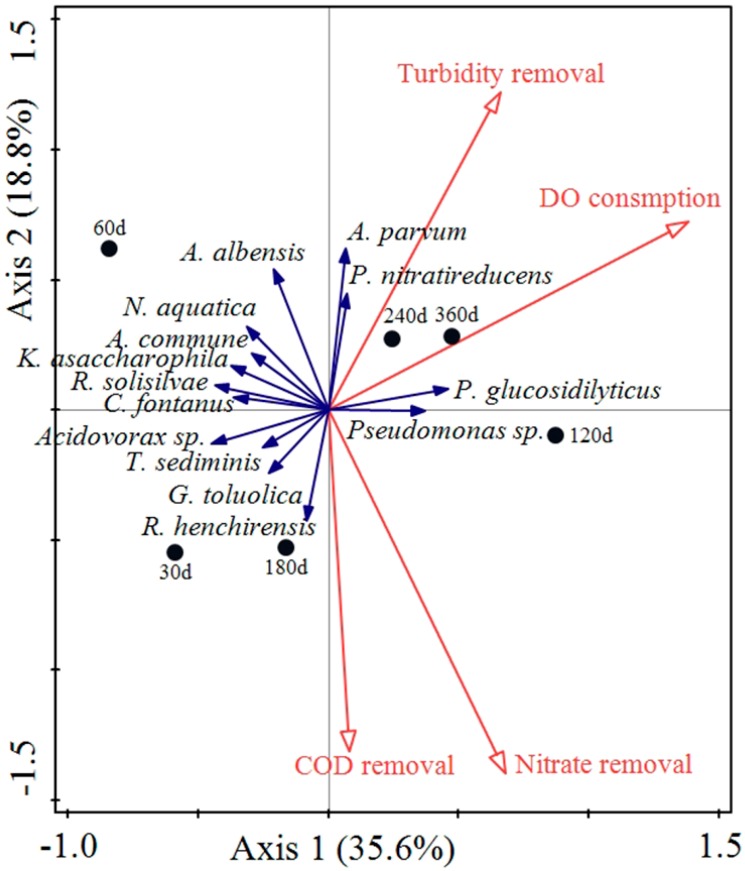
Redundancy analysis (RDA) ordination diagram of identified species (blue arrows) and filter performance (red arrows).

A redundancy analysis (RDA) was performed to assess the possible correlations between the identified bacterial species and the pilot-scale filter performance (shown in Figure 6). Parameters including COD_Mn_ removal, turbidity removal, nitrate-N removal, and DO consumption were analyzed for the 14 identified species in 6 samples at different running times. Arithmetic mean values were used to present the final abundances of different bacteria in the BAC filter. Based on the RDA results, *Ramlibacter henchirensis* and *Georgfuchsia toluolica* were positively correlated to COD_Mn_ removal, and *Ramlibacter henchirensis* showed a positive correlation with nitrate-N removal. *Aquabacterium parvum* and *Phaeobacterium nitratireducens* were associated with turbidity removal. *Pedobacter glucosidilyticus* and *Pseudomonas sp.* were positively correlated with high DO consumption.

Among these bacteria, *Ramlibacter henchirensis* was reported as an aerobic chemo-organotrophic and slow-growing betaproteobacterium, and as positive for the reduction of nitrate to nitrite. It was detected in the aerobic tanks of different lab-scale wastewater treatment bioreactors. A wide range of metabolic ability was suggested for organic and nitrate substrates under a long retention time [18,19]. *Georgfuchsia toluolica* was once isolated from a polluted aquifer, and it was described as an anaerobic betaproteobacterium which could degrade a series of aromatic compounds [29]. *Aquabacterium parvum* was found as a dominant bacterium from biofilms in the Berlin drinking water system, and its characterization as a nitrate-dependent Fe(II)-oxidizing (NDFO) bacterium is well known [30,31]. *Phaeobacterium nitratireducens* was reported as a photo-organoheterotrophic, purple sulfur gammaproteobacterium [28]. The correlations between these two species and turbidity removal are still not well-documented. *Pedobacter glucosidilyticus* was described as an aerobic, rod-shaped gliding bacterium. With specific physiological properties of phosphite assimilation and phosphonoacetate utilization, *Pedobacter glucosidilyticus* participated in the phosphorus metabolism of the bacterial community [32,33]. The microorganisms of the genus *Pseudomonas* are usually strict aerobic betaproteobacteria, having swimming motility via polar flagella, and are widely distributed in environments such as soil, water, and the skin of animals (including humans). The correlation with the high DO consumption indicates a strong oxidative metabolism. Other species were weakly associated with any examined performance parameters.

## 4. Discussion

In this study, a pilot-scale filter of a O_3_/BAC process operated over 360 days. As the results above describe, a preliminary model of the bacterial community succession was outlined for the first time. The period from 0 to 60 days was the set-up stage of the BAC filter, in which bacteria from influent water rapidly aggregated in the activated carbon surface area and in well-developed pores by physical adsorption, and formed an initial community. A few advantageous species from orders burkholderiales and nitrosomonadales, i.e., *Curvibacter fontanus*, *Kinneretia asaccharophila*, *Tibeticola sediminis,* and *Georgfuchsia toluolica,* reproduced and enriched rapidly in all activated carbon layers, and in total accounted for up to 52.8% of the bacterial community. Subsequently, a fluctuation stage was observed from day 61 to 120, in which a serious shift of the bacterial community was caused by later bacteria brought in with the constant influent water. The diversity of the community increased slightly, whereas the adjacent similarity of the activated carbon layers reduced, and the competition among species intensified. The relative abundances of unadaptable species, including *Ramlibacter solisilvae* and *Ramlibacter henchirensis*, decreased and vanished in the filter during this period. As the competitive species survived and dominated, the BAC filter entered a stable stage during days 121 to 360. The abundances of dominant species equalized, and the bacterial community diversity further increased. Eventually, the bacterial community attached on the activated carbon matured in order, from bottom to top.

In the drinking water treatment, the primary dynamic pattern was the variation of bacterial communities with treatment processes, rather than temporal fluctuation [13]. The bacterial communities gradually matured and stabilized with the filter running. In that regard, a complex bacterial community with a more diverse set of dominant species has been reported in an investigation on a full-scale O_3_/BAC filter [34]. In addition, α-Proteobacteria, β-Proteobacteria, γ-Proteobacteria, Bacteroidetes, Actinobacteria, Firmicutes, and Cyanobacteria were revealed as the dominant bacteria groups in the effluent of the BAC filter. The difference may be caused by the longer filter operation time, or by the dominant species in the upstream treatment processes. As compared with previous studies, the Proteobacteria and Bacteroidetes were also advantageous bacterial phyla during the occurrence of the procedure. It is reported that Proteobacteria exhibit a competitive advantage in a drinking water treatment with low BDOC [35]. Bacteroidetes is commonly observed in soil ecosystems, but its ecological role is still unclear, partly owing to the difficulties in culturing it [35].

In this research, a series of bacteria correlated with filter performance were detected, including *Ramlibacter henchirensis*, *Georgfuchsia toluolica*, *Aquabacterium parvum*, *Phaeobacterium nitratireducens*, *Pedobacter glucosidilyticus*, and *Pseudomonas sp.* Meanwhile, no OP or potential pathogen was observed, indicating that the relevant species were less likely to dominate during the occurrence of the bacteria community. However, it should be considered as a preliminary result, as limited biological information was obtained through PCR-DGGE. Thus, a metagenome analysis with a high-throughput sequencing method is suggested for a full-scale O_3_/BAC drinking water treatment plant.

## 5. Conclusions

The main conclusions of this research are summarized as follows:

(1) After a preliminary bacterial community initially formed, the species abundance and diversity of the bacterial communities attached to the activated carbon increased with the study duration. A succession procedure of the bacterial community was observed, including a set-up stage from day 0 to 60, a fluctuation stage from day 61 to 120, and a stable stage from day 121 to 360. The bacterial community was dominated by Proteobacteria and Bacteroidetes.
(2) PCA indicated that the bacterial communities in different layers clustered and tended to be consistent after 60 days. The lower activated carbon layers proved to be more conducive to the stability and maturation of the bacterial community under sufficient DO conditions.
(3) The average COD_Mn_, turbidity removal rate, and DO consumption rate for the BAC filter were 26.43%, 16.57%, and 16.4%, respectively.
(4) RDA revealed that *Ramlibacter henchirensis* was positively correlated to COD_Mn_ removal and nitrate-N removal. *Georgfuchsia toluolica* showed a positive correlation with COD_Mn_ removal. *Aquabacterium parvum* and *Phaeobacterium nitratireducens* were positively associated with turbidity removal. *Pedobacter glucosidilyticus* and *Pseudomonas sp*. were associated with a high DO consumption.

## Figures and Tables

**Table 1:** Performance parameters of activated carbon.

Parameter	Value	Unit
Tannic Value	756	mg/L
Surface area	1086	m^2^/g
Volume of pore	0.57	cm^3^/g
Iodine number	988	mg/g
Methylene blue	180	mg/g

**Table 2:** Main requirements of standards for drinking water quality GB5749-2006.

Parameter	Requirement	Unit
COD_Mn_	≤3	mg/L
Nitrate nitrogen	≤10	mg/L
Turbidity	≤1	NTU
pH	6.5 to 8.5	-

**Table 3:** Identification of dominant bacterial species by sequence blast in GeneBank.

Band No.	GeneBank Accession No.	Nearest Relative Species of 16S rRNA Blast (Reference GeneBank Accession No.)	Similarity Index (%)
Band 1	MH973208	*Pseudomonas sp.* (NR_117678.1)	99
Band 2	MH973209	*Acinetobacter albensis* (NR_145641.1)	98
Band 3	MH973210	*Pedobacter glucosidilyticus* (NR_116376.1)	98
Band 4	MH973211	*Niabella aquatica* (NR_151879.1)	93
Band 5	MH973212	*Kinneretia asaccharophila* (NR_115151.1)	96
Band 6	MH973213	*Aquabacterium parvum* (NR_024874.1)	99
Band 7	MH973214	*Georgfuchsia toluolica* (NR_115995.1)	92
Band 8	MH973215	*Tibeticola sediminis* (NR_156927.1)	96
Band 9	MH973216	*Curvibacter fontanus* (NR_112221.1)	99
Band 10	MH973217	*Phaeobacterium nitratireducens* (NR_136764.1)	95
Band 11	MH973218	*Ramlibacter solisilvae* (NR_133837.1)	94
Band 12	MH973219	*Aquabacterium commune* (NR_024875.1)	97
Band 13	MH973220	*Ramlibacter henchirensis* (NR_025203.1)	94
Band 14	MH973221	*Acidovorax sp.* (NR_116740.1)	97

**Table 4:** Operational taxonomic units (OTUs) and Shannon index of different samples.

Time	OTUs ^1^			Shannon Index ^2^		
	0.25 m Depth	0.75 m Depth	1.25 m Depth	0.25 m Depth	0.75 m Depth	1.25 m Depth
30 d	16	23	19	2.52	2.91	2.70
60 d	16	24	23	2.60	3.05	2.98
120 d	18	24	30 ^3^	2.82	3.07	3.28 ^3^
180 d	16	30 ^3^	27	2.60	3.30^3^	3.15
240 d	25 ^3^	30	31	3.09 ^3^	3.27	3.27
360 d	31	34	30	3.33	3.43	3.30

^1^ OTUs: a higher number indicates more bacterial species. ^2^ Shannon index: a higher number indicates more diversity of community. ^3^ Intense increase of OTUs or Shannon index.
